# Supplementary material for: A Likelihood Approach for Real-Time Calibration of Stochastic Compartmental Epidemic Models
Source: PLoS Comput Biol. 2017 Jan 17;13(1):e1005257. doi: 10.1371/journal.pcbi.1005257 (PMC5240920; doi:10.1371/journal.pcbi.1005257)
Supplement: S1 File — (TAR.GZ) [file pcbi.1005257.s014.tar.gz › HSPH_Online-SI-Revision/EnKF07/n1-severe/n1-severe_EnKF2_table0.pdf]

1	newly 3 weeks spec	27.	10.59	{0., 13.}	101.815	1.33553	newly 3 weeks cumul	92.	94.48	{8., 87.}	114.891	0.547316	newly 5 weeks spec	28.	3.06	{0., 7.}	91.9286	1.81722	newly 5 weeks cumul	149.	102.61	{8., 99.}	102.691	0.686615	newly 54 weeks cumul	407.	108.53	{8., 149.}	93.0688	1.0461
2	newly 3 weeks spec	51.	14.97	{0., 25.}	94.1765	1.34472	newly 3 weeks cumul	151.	111.5	{9., 311.}	107.06	0.744747	newly 5 weeks spec	43.	5.71	{0., 9.}	95.3721	1.92719	newly 5 weeks cumul	237.	125.73	{11., 311.}	97.3797	0.856234	newly 56 weeks cumul	398.	139.31	{11., 313.}	91.0477	1.01376
3	newly 3 weeks spec	41.	18.47	{0., 18.}	109.976	1.39285	newly 3 weeks cumul	126.	110.8	{6., 197.}	122.889	0.801873	newly 5 weeks spec	34.	7.5	{0., 11.}	95.	1.71934	newly 5 weeks cumul	205.	129.68	{6., 283.}	110.127	0.924587	newly 55 weeks cumul	432.	144.17	{7., 449.}	93.7616	1.14684
4	newly 3 weeks spec	70.	8.18	{0., 16.}	91.1143	1.71789	newly 3 weeks cumul	194.	98.87	{10., 109.}	93.2526	0.757985	newly 5 weeks spec	40.	2.46	{0., 6.}	94.85	1.98304	newly 5 weeks cumul	270.	105.96	{10., 133.}	89.8815	0.844982	newly 58 weeks cumul	393.	110.12	{10., 138.}	87.6692	0.965053
5	newly 3 weeks spec	50.	8.7	{0., 20.}	93.48	1.63912	newly 3 weeks cumul	135.	104.8	{6., 120.}	114.978	0.743966	newly 5 weeks spec	19.	3.86	{0., 9.}	93.6842	1.58662	newly 5 weeks cumul	204.	114.55	{7., 157.}	104.123	0.84613	newly 57 weeks cumul	333.	124.18	{7., 180.}	95.2372	0.980477
6	newly 3 weeks spec	37.	10.49	{0., 14.}	95.4324	1.37421	newly 3 weeks cumul	122.	80.86	{12., 113.}	110.656	0.759885	newly 5 weeks spec	35.	4.81	{0., 6.}	97.1143	1.90184	newly 5 weeks cumul	196.	92.17	{13., 262.}	100.046	0.876258	newly 54 weeks cumul	501.	115.04	{13., 352.}	89.976	1.17019
7	newly 3 weeks spec	30.	5.78	{0., 10.}	91.2	1.38217	newly 3 weeks cumul	90.	76.52	{10., 136.}	114.8	0.664926	newly 5 weeks spec	29.	2.58	{0., 3.}	96.069	1.91533	newly 5 weeks cumul	156.	82.76	{13., 236.}	100.5	0.797874	newly 54 weeks cumul	252.	90.84	{14., 270.}	94.0317	0.94646
8	newly 3 weeks spec	38.	9.71	{0., 17.}	94.1316	1.45947	newly 3 weeks cumul	207.	105.75	{14., 131.}	91.6667	0.723295	newly 5 weeks spec	30.	3.5	{0., 8.}	93.3333	1.83013	newly 5 weeks cumul	276.	114.71	{14., 154.}	89.5036	0.797533	newly 58 weeks cumul	301.	120.2	{14., 155.}	88.5914	0.810538
9	newly 3 weeks spec	23.	8.11	{0., 10.}	97.7826	1.18019	newly 3 weeks cumul	75.	82.63	{14., 230.}	129.587	0.5693	newly 5 weeks spec	38.	2.67	{0., 5.}	95.3421	1.94216	newly 5 weeks cumul	137.	89.67	{16., 253.}	107.015	0.729519	newly 54 weeks cumul	335.	96.39	{17., 260.}	89.2866	0.997789
10	newly 3 weeks spec	42.	24.29	{0., 38.}	118.976	1.30905	newly 3 weeks cumul	146.	114.95	{12., 473.}	119.185	0.79274	newly 5 weeks spec	28.	8.89	{0., 12.}	102.679	1.6155	newly 5 weeks cumul	205.	139.14	{15., 650.}	113.795	0.856618	newly 54 weeks cumul	314.	152.77	{19., 698.}	101.634	0.957379
11	newly 3 weeks spec	26.	3.29	{0., 7.}	96.5	2.00039	newly 3 weeks cumul	86.	56.9	{7., 110.}	77.4419	0.536551	newly 5 weeks spec	24.	2.64	{0., 1.}	102.25	2.19806	newly 5 weeks cumul	140.	63.02	{7., 141.}	80.7286	0.70102	newly 51 weeks cumul	390.	75.36	{7., 141.}	88.041	1.08973
12	newly 3 weeks spec	37.	21.05	{0., 85.}	112.027	1.25102	newly 3 weeks cumul	137.	146.8	{10., 448.}	132.803	0.710518	newly 5 weeks spec	21.	8.7	{0., 8.}	113.524	1.68623	newly 5 weeks cumul	184.	167.94	{10., 717.}	124.5	0.784696	newly 59 weeks cumul	237.	185.51	{10., 827.}	117.498	0.850943
13	newly 3 weeks spec	52.	11.97	{0., 12.}	97.75	1.50247	newly 3 weeks cumul	157.	77.11	{15., 52.}	100.58	0.804799	newly 5 weeks spec	31.	3.84	{0., 4.}	96.129	1.88122	newly 5 weeks cumul	230.	87.17	{18., 131.}	95.9435	0.894736	newly 54 weeks cumul	413.	102.2	{19., 199.}	90.799	1.06716
14	newly 3 weeks spec	54.	23.79	{0., 85.}	104.241	1.43978	newly 3 weeks cumul	204.	157.87	{8., 703.}	116.515	0.88525	newly 5 weeks spec	35.	6.94	{0., 12.}	94.8	1.89453	newly 5 weeks cumul	282.	177.71	{8., 919.}	108.032	0.966189	newly 59 weeks cumul	417.	190.43	{8., 937.}	97.2254	1.07991
15	newly 3 weeks spec	15.	16.1	{0., 19.}	146.267	1.02688	newly 3 weeks cumul	83.	82.67	{9., 77.}	117.434	0.573431	newly 5 weeks spec	28.	5.6	{0., 15.}	93.7857	1.70208	newly 5 weeks cumul	134.	97.85	{10., 137.}	108.261	0.702361	newly 53 weeks cumul	180.	106.92	{10., 275.}	102.478	0.790807
16	newly 3 weeks spec	53.	21.08	{0., 67.}	100.302	1.30393	newly 3 weeks cumul	165.	153.99	{7., 698.}	121.994	0.747647	newly 5 weeks spec	40.	8.01	{0., 16.}	94.225	1.81486	newly 5 weeks cumul	245.	174.16	{8., 809.}	108.49	0.837069	newly 59 weeks cumul	406.	189.19	{8., 839.}	93.697	0.97474
17	newly 3 weeks spec	52.	20.29	{0., 62.}	98.5577	1.3352	newly 3 weeks cumul	182.	132.37	{12., 231.}	102.577	0.728972	newly 5 weeks spec	22.	8.59	{0., 18.}	104.591	1.55852	newly 5 weeks cumul	246.	154.61	{14., 417.}	99.2398	0.798236	newly 59 weeks cumul	311.	179.9	{15., 791.}	98.7138	0.859023
18	newly 3 weeks spec	25.	13.07	{0., 17.}	104.04	1.07244	newly 3 weeks cumul	80.	91.32	{11., 124.}	111.675	0.484371	newly 5 weeks spec	24.	3.81	{0., 7.}	93.2083	1.68574	newly 5 weeks cumul	126.	102.19	{13., 188.}	101.325	0.605024	newly 59 weeks cumul	490.	115.68	{15., 219.}	87.7469	1.06755
19	newly 3 weeks spec	33.	9.45	{1., 11.}	89.303	0.942987	newly 3 weeks cumul	111.	54.88	{14., 60.}	79.7838	0.573659	newly 5 weeks spec	35.	3.15	{0., 6.}	95.6286	1.73597	newly 5 weeks cumul	180.	63.63	{17., 67.}	84.55	0.714236	newly 52 weeks cumul	331.	71.13	{25., 73.}	87.6465	0.91245
20	newly 3 weeks spec	32.	3.17	{0., 7.}	92.6563	1.72227	newly 3 weeks cumul	124.	87.69	{9., 110.}	102.702	0.683551	newly 5 weeks spec	27.	0.59	{0., 2.}	97.8148	2.16155	newly 5 weeks cumul	188.	89.2	{9., 110.}	95.1064	0.818789	newly 52 weeks cumul	258.	90.52	{9., 112.}	91.062	0.921322
21	newly 3 weeks spec	34.	21.24	{0., 66.}	114.235	1.29453	newly 3 weeks cumul	127.	150.23	{12., 515.}	129.299	0.599374	newly 5 weeks spec	35.	9.76	{0., 21.}	98.2857	1.70195	newly 5 weeks cumul	201.	172.87	{12., 788.}	113.08	0.709363	newly 59 weeks cumul	389.	192.12	{12., 838.}	94.5964	0.89527
22	newly 3 weeks spec	28.	10.08	{0., 9.}	108.357	1.50531	newly 3 weeks cumul	132.	97.08	{9., 92.}	111.697	0.74946	newly 5 weeks spec	29.	1.05	{0., 2.}	96.3793	2.21773	newly 5 weeks cumul	187.	101.27	{11., 105.}	103.332	0.859384	newly 53 weeks cumul	239.	102.13	{11., 122.}	98.4728	0.940931
23	newly 3 weeks spec	28.	10.41	{0., 23.}	89.8929	1.12178	newly 3 weeks cumul	106.	99.83	{8., 178.}	118.274	0.641111	newly 5 weeks spec	31.	2.16	{0., 8.}	93.0323	1.88459	newly 5 weeks cumul	167.	106.41	{9., 198.}	103.527	0.764814	newly 55 weeks cumul	324.	109.83	{9., 223.}	92.1698	0.979957
24	newly 3 weeks spec	36.	11.3	{0., 14.}	92.4444	1.01277	newly 3 weeks cumul	101.	68.63	{16., 78.}	86.3069	0.529049	newly 5 weeks spec	23.	4.83	{0., 7.}	94.3043	1.50045	newly 5 weeks cumul	158.	80.52	{19., 82.}	87.1266	0.642357	newly 52 weeks cumul	238.	105.46	{22., 96.}	90.5378	0.754594
25	newly 3 weeks spec	53.	11.32	{0., 27.}	90.4151	1.58062	newly 3 weeks cumul	164.	111.91	{15., 265.}	98.0793	0.673368	newly 5 weeks spec	31.	3.03	{0., 7.}	94.9355	1.96854	newly 5 weeks cumul	226.	119.84	{15., 269.}	91.6018	0.75116	newly 50 weeks cumul	295.	127.76	{15., 307.}	86.9017	0.814207
26	newly 3 weeks spec	42.	6.46	{0., 11.}	92.381	1.60811	newly 3 weeks cumul	125.	74.91	{5., 99.}	102.84	0.806586	newly 5 weeks spec	18.	2.9	{0., 8.}	94.6667	1.75341	newly 5 weeks cumul	181.	82.09	{5., 133.}	97.5083	0.902095	newly 57 weeks cumul	332.	90.35	{5., 139.}	93.997	1.10577
27	newly 3 weeks spec	52.	13.87	{0., 41.}	82.8269	1.19292	newly 3 weeks cumul	169.	104.41	{12., 266.}	93.8876	0.718149	newly 5 weeks spec	14.	5.95	{0., 15.}	94.0714	1.31079	newly 5 weeks cumul	215.	118.09	{12., 288.}	88.2465	0.741201	newly 56 weeks cumul	229.	144.14	{12., 329.}	89.607	0.72967
28	newly 3 weeks spec	51.	19.22	{0., 53.}	100.588	1.36382	newly 3 weeks cumul	140.	134.6	{8., 316.}	123.314	0.729976	newly 5 weeks spec	48.	8.41	{0., 12.}	95.6042	1.93975	newly 5 weeks cumul	238.	155.49	{8., 477.}	105.416	0.863498	newly 59 weeks cumul	446.	170.75	{8., 721.}	91.1278	1.04791
29	newly 3 weeks spec	34.	14.75	{0., 45.}	89.0294	1.03505	newly 3 weeks cumul	114.	128.41	{5., 488.}	125.149	0.625117	newly 5 weeks spec	17.	3.28	{0., 8.}	89.4118	1.46014	newly 5 weeks cumul	151.	138.51	{5., 534.}	112.616	0.679125	newly 52 weeks cumul	284.	143.32	{5., 542.}	92.5563	0.862493
30	newly 3 weeks spec	37.	11.89	{0., 22.}	93.3243	1.48992	newly 3 weeks cumul	120.	101.16	{12., 138.}	89.85	0.536813	newly 5 weeks spec	28.	5.08	{0., 9.}	95.5	1.77669	newly 5 weeks cumul	177.	113.23	{12., 152.}	87.4859	0.642722	newly 53 weeks cumul	261.	126.22	{12., 213.}	86.2912	0.759179
31	newly 3 weeks spec	31.	12.13	{0., 23.}	99.9032	1.28407	newly 3 weeks cumul	87.	101.35	{7., 103.}	136.103	0.614038	newly 5 weeks spec	16.	2.63	{0., 4.}	95.3125	1.71516	newly 5 weeks cumul	126.	109.14	{7., 152.}	120.476	0.71963	newly 54 weeks cumul	328.	114.46	{7., 206.}	95.1707	1.03901
32	newly 3 weeks spec	17.	6.81	{0., 19.}	81.8235	1.20814	newly 3 weeks cumul	59.	78.81	{8., 119.}	129.068	0.501344	newly 5 weeks spec	26.	3.94	{0., 9.}	92.5385	1.80859	newly 5 weeks cumul	103.	87.31	{8., 142.}	104.922	0.646364	newly 56 weeks cumul	357.	111.59	{8., 265.}	89.0672	1.0512
33	newly 3 weeks spec	51.	18.43	{0., 68.}	94.4902	1.34894	newly 3 weeks cumul	172.	127.36	{8., 247.}	104.337	0.752502	newly 5 weeks spec	50.	7.89	{0., 18.}	93.14	1.96306	newly 5 weeks cumul	284.	146.9	{8., 403.}	94.6127	0.886565	newly 59 weeks cumul	418.	169.09	{8., 760.}	90.1746	0.996113
34	newly 3 weeks spec	13.	24.62	{1., 61.}	177.077	0.593253	newly 3 weeks cumul	56.	163.95	{27., 392.}	222.768	0.33764	newly 5 weeks spec	29.	6.29	{0., 15.}	91.	1.54158	newly 5 weeks cumul	105.	182.12	{27., 446.}	144.705	0.402497	newly 51 weeks cumul	255.	190.36	{27., 488.}	97.3333	0.624655
35	newly 3 weeks spec	56.	23.84	{0., 81.}	100.607	1.38288	newly 3 weeks cumul	196.	144.86	{8., 335.}	111.908	0.866989	newly 5 weeks spec	34.	7.8	{0., 18.}	94.5294	1.72016	newly 5 weeks cumul	269.	165.48	{8., 411.}	104.349	0.93267	newly 59 weeks cumul	333.	183.2	{10., 777.}	99.6817	0.978993
36	newly 3 weeks spec	35.	19.09	{0., 52.}	105.629	1.26891	newly 3 weeks cumul	137.	105.08	{15., 280.}	84.2628	0.531905	newly 5 weeks spec	9.	7.71	{0., 10.}	131.	1.24535	newly 5 weeks cumul	166.	125.14	{17., 320.}	89.6386	0.575265	newly 60 weeks cumul	168.	136.9	{17., 346.}	94.3452	0.575341
37	newly 3 weeks spec	30.	5.	{0., 12.}	90.3333	1.76754	newly 3 weeks cumul	103.	118.53	{4., 312.}	148.379	0.816999	newly 5 weeks spec	13.	0.49	{0., 1.}	96.2308	1.93073	newly 5 weeks cumul	136.	120.54	{4., 312.}	129.956	0.893379	newly 50 weeks cumul	294.	120.82	{4., 312.}	100.184	1.14013
38	newly 3 weeks spec	36.	8.57	{0., 19.}	87.0833	1.33793	newly 3 weeks cumul	146.	121.84	{10., 134.}	105.397	0.631926	newly 5 weeks spec	22.	2.23	{0., 7.}	91.4091	1.72024	newly 5 weeks cumul	199.	128.62	{10., 164.}	98.0302	0.710718	newly 58 weeks cumul	228.	132.42	{10., 179.}	94.9649	0.740057
39	newly 3 weeks spec	27.	7.76	{0., 20.}	88.2222	1.26587	newly 3 weeks cumul	108.	71.05	{7., 198.}	99.6759	0.717941	newly 5 weeks spec	28.	2.29	{0., 6.}	91.8214	1.84686	newly 5 weeks cumul	158.	77.29	{8., 199.}	92.3734	0.809369	newly 54 weeks cumul	281.	82.32	{8., 247.}	85.4733	0.977049
40	newly 3 weeks spec	44.	17.85	{0., 47.}	93.6591	1.29072	newly 3 weeks cumul	123.	143.64	{15., 412.}	110.959	0.494004	newly 5 weeks spec	23.	7.17	{0., 16.}	97.2609	1.63615	newly 5 weeks cumul	179.	162.24	{20., 459.}	100.268	0.568733	newly 60 weeks cumul	274.	176.51	{21., 526.}	90.8723	0.680899
41	newly 3 weeks spec	39.	9.42	{0., 15.}	93.8462	1.55048	newly 3 weeks cumul	166.	81.91	{10., 119.}	93.8735	0.788263	newly 5 weeks spec	19.	5.58	{0., 10.}	96.8421	1.54609	newly 5 weeks cumul	210.	95.23	{12., 149.}	92.0429	0.82137	newly 57 weeks cumul	257.	106.67	{12., 182.}	89.2724	0.849411
42	newly 3 weeks spec	38.	13.63	{0., 20.}	96.8158	1.33967	newly 3 weeks cumul	107.	91.31	{13., 122.}	94.8692	0.5365	newly 5 weeks spec	31.	5.37	{0., 9.}	97.129	1.81119	newly 5 weeks cumul	165.	105.36	{14., 159.}	91.9273	0.651806	newly 57 weeks cumul	391.	118.8	{14., 204.}	87.202	0.937607
43	newly 3 weeks spec	32.	4.17	{0., 12.}	92.1563	1.90249	newly 3 weeks cumul	103.	64.45	{3., 71.}	108.165	0.846838	newly 5 weeks spec	12.	1.01	{0., 2.}	93.0833	1.82401	newly 5 weeks cumul	138.	67.56	{3., 99.}	101.58	0.938365	newly 50 weeks cumul	246.	70.47	{3., 136.}	93.0528	1.13294
44	newly 3 weeks spec	38.	10.9	{0., 17.}	93.5789	1.45224	newly 3 weeks cumul	148.	95.38	{13., 109.}	94.9324	0.660221	newly 5 weeks spec	25.	3.72	{0., 6.}	95.04	1.82089	newly 5 weeks cumul	218.	104.8	{15., 128.}	92.055	0.776228	newly 58 weeks cumul	302.	110.56	{15., 135.}	89.5497	0.879719
45	newly 3 weeks spec	39.	12.3	{0., 31.}	87.2821	1.20564	newly 3 weeks cumul	137.	111.92	{12., 383.}	102.526	0.628768	newly 5 weeks spec	24.	3.98	{0., 6.}	96.25	1.73783	newly 5 weeks cumul	197.	122.03	{14., 431.}	93.5482	0.705858	newly 55 weeks cumul	330.	128.53	{15., 431.}	84.9788	0.852594
46	newly 3 weeks spec	24.	4.49	{0., 9.}	88.625	1.27216	newly 3 weeks cumul	77.	65.14	{16., 43.}	111.325	0.56324	newly 5 weeks spec	26.	2.68	{0., 6.}	93.6923	1.83789	newly 5 weeks cumul	128.	71.13	{18., 56.}	99.2734	0.702447	newly 54 weeks cumul	420.	88.72	{18., 96.}	89.2095	1.10415
47	newly 3 weeks spec	53.	20.91	{0., 73.}	90.434	1.20061	newly 3 weeks cumul	225.	138.36	{17., 350.}	83.4933	0.626265	newly 5 weeks spec	15.	10.99	{0., 31.}	126.733	1.39317	newly 5 weeks cumul	267.	164.4	{18., 443.}	85.2809	0.662152	newly 60 weeks cumul	272.	183.19	{20., 503.}	89.7463	0.666599
48	newly 3 weeks spec	21.	7.71	{0., 11.}	87.6667	1.00732	newly 3 weeks cumul	100.	71.6	{17., 102.}	93.28	0.550836	newly 5 weeks spec	11.	2.43	{0., 5.}	88.4545	1.336	newly 5 weeks cumul	125.	77.86	{17., 103.}	89.648	0.584898	newly 54 weeks cumul	216.	82.74	{17., 142.}	85.4907	0.749563
49	newly 3 weeks spec	40.	11.94	{0., 18.}	97.7	1.46304	newly 3 weeks cumul	144.	105.35	{15., 125.}	98.9236	0.648209	newly 5 weeks spec	31.	3.72	{0., 8.}	96.2581	1.94584	newly 5 weeks cumul	205.	115.09	{15., 141.}	94.7171	0.748531	newly 58 weeks cumul	301.	119.96	{15., 157.}	90.3654	0.871872
50	newly 3 weeks spec	29.	11.71	{0., 19.}	100.517	1.43454	newly 3 weeks cumul	126.	75.28	{5., 120.}	92.5556	0.733706	newly 5 weeks spec	18.	4.88	{0., 10.}	101.889	1.80037	newly 5 weeks cumul	161.	86.98	{5., 197.}	93.3292	0.795348	newly 56 weeks cumul	315.	96.8	{5., 197.}	88.5397	1.01659
